# “Living in a Communal Garden” Associated with Well-Being While Reducing Urban Sprawl by 40%: A Mixed-Methods Cross-Sectional Study

**DOI:** 10.3389/fpubh.2015.00173

**Published:** 2015-07-20

**Authors:** Jamie Anderson

**Affiliations:** ^1^Cambridge Well-Being Institute, University of Cambridge, Cambridge, UK; ^2^Social Life (Social Enterprise Company), London, UK; ^3^Department of Architecture, University of Cambridge, Cambridge, UK

**Keywords:** well-being, health behaviors, neighborhood, public space, urban sprawl

## Abstract

**Background:**

The extent to which novel land-efficient neighborhood design can promote key health behaviors is examined, concentrating on communal outdoor space provision (COSP).

**Objectives:**

To test whether a neighborhood (Accordia) with a higher ratio of communal to private outdoor space is associated with higher levels of resident’s (a) self-reported local health behaviors and (b) observed engagement in local health behaviors, compared to a matched neighborhood with lower proportion of COSP.

**Methods:**

Health behaviors were examined via direct observation and postal survey. Bespoke observation codes and survey items represented key well-being behaviors including “connecting,” “keeping active,” “taking notice,” “keep learning,” and “giving.” The questionnaire was validated using psychometric analyses and observed behaviors were mapped in real-time.

**Results:**

General pursuit of health behaviors was very similar in both areas but Accordia residents reported substantially greater levels of local activity. Validated testing of survey dataset (*n* = 256) showed support for a stronger Attitude to Neighborhood Life (connecting and giving locally) in Accordia and partial support of greater physical activity. Analyses of the behavior observation dataset (*n* = 7,298) support the self-reported findings. Mapped observations revealed a proliferation of activity within Accordia’s innovative outdoor *hard* spaces.

**Conclusion:**

Representation is limited to upper-middle class UK groups. However, Accordia was found to promote health behaviors compared a traditional neighborhood that demands considerably more land area. The positive role of *home zone* streets, hard-standing and *semi-civic* space highlights the principle of quality as well as quantity. The findings should be considered as part of three forthcoming locally led UK garden cities, to be built before 2020.

## Introduction

The previous UK coalition Government announced the building of three *garden cities* before 2020 (1). Within its prospectus, the Government states that they do not wish to impose a definition of what garden cities are. However, among other principles, it is set out that local authorities may consider the inclusion of private gardens, generous green space, and allotments, while avoiding *urban sprawl* (1). Within this study, the idea of reduced private garden space, in proportion to communal outdoor space provision (COSP), is tested as a way to promote healthy and vibrant communities, while reducing encroachment on countryside and green belt.

It is claimed that early pioneers – such as Ebenezer Howard’s garden city movement, were visionary in their ambitions for high well-being communities (2) and several garden city principles are associated with activities that are good for health. Positive features include amount of green space in relation to walking for pleasure and physical activity, children’s play, and socializing (3, 4). However, the current evidence base thus far often relies upon non-validated measures, self-reported findings, and a distinct lack of experimental studies (5, 6). Limited research has demonstrated the perceived importance of private gardens for well-being, particularly among older people (7). A wider body of research has begun to show that communal gardens can have beneficial impacts for well-being, in the UK (8) and beyond (9). Well-being insight pertaining to urban and hard-landscaped types of public space, such as squares and boulevards, is narrow (5).

Drawing upon the “Five-Ways to Well-being” activity framework and a mixed-methods approach, this study focuses on the question: does a neighborhood with more communal than private green space promote *Five-Ways* health behaviors among local residents? The research also asks the question: which *types* of outdoor civic space are observed to be the most popular among residents?

The Five-Ways were identified by the New Economics Foundation (*nef*) when summarizing evidence on well-being, produced as part of the Foresight Report (2008) on Mental Capital and Well-being (10, 11). The Five-Ways comprises (a) Connect, (b) Keep Active, (c) Take Notice, (d) Keep Learning, and (e) Give. Quantity and quality of individual social connections are critically correlated with subjective well-being (SWB) (12–16). Being physically active is associated with higher SWB and the prevention of a range of chronic physical diseases (17, 18). Taking notice (or mindfulness) has been strongly linked with higher SWB and fewer negative symptoms, such as anxiety or depression (19–21). Continued learning though life is associated with SWB and cognitive development (22–24) and altruistic experiments and large scale observational studies have strongly linked pro-social activity with happiness (25, 26).

In summary, there is no lack of evidence showing why the Five-Ways activities are important for health and high SWB. This research builds on this evidence by demonstrating how the limited provision of private gardens, in proportion to COSP, may be linked to several domains within the Five-Ways activity framework.

### Objectives

The primary objective of the study is to determine whether the prevalence of Five-Ways health behaviors is associated with a higher COSP among upper-middle-class residents in Cambridge (UK). The secondary objective is to identify specific types of COSP associated with observed behaviors.

## Materials and Methods

### Study design

The research comprised a mixed-methods cross-sectional study undertaken in 2012. The prevalence of health behaviors was measured in a neighborhood with few private gardens but, has a high volume of COSP, and a second area where most houses have private gardens (but a relatively low amount of local COSP). Both areas comprised predominantly upper-middle class residents and had similar general urban design characteristics. The analysis not only concentrates on amount of COSP but also considers four key types of COSP: green space, play and sports areas, *hard-civic*, and *semi-civic* spaces. Although these types of space are often referred to by urban designers and town planners, their significance in terms of their effect on human health and well-being is not adequately understood. The two areas were compared using both self-report and direct observation datasets. As well as residents reporting on their activity, residents behavior was also observed directly as they went about their neighborhood day-to-day outdoor activities. The behaviors were mapped so that the results can be more clearly related to specific types of COSP. Differences between neighborhoods and differences between datasets were compared, taking important confounding and biases into account.

### Setting

The neighborhood with abundant COSP is named Accordia. The design of this neighborhood was driven by a unifying idea of “living in a garden” (27). Considerable native landscape was integrated into the design and a very small number of trees were felled, so that Accordia boasts more than 700 mature trees. Perhaps, the most radical aspect of the design is that only around 25% of the residents have a private garden. Instead, many homes have access to interior terraces on the first and/or second floors, internal courtyards, or semi-private community gardens. They also enjoy good access to countryside via an adjacent green corridor (see Supplementary Material). However, within the neighborhood itself and as shown in Table 1, all Accordia residents share 3.8 hectares of COSP, which represents approximately 39% of this land area. In contrast, this area was compared to a neighborhood in Castle that comprises 1.8 hectares of COSP, around 11% of the total area.

**Table 1:** **Summary comparison of COSP in Accordia and Castle**.

	Accordia		Castle	
	hec	%	hec	%
COSP	3.8	39	1.8	11
Private outdoor space	0.6	5	7.3	44
COSP types				
1. Green space	2.6	28	1.4	9
2. Play and sports	0.2	1.7	0.4	2.2
3. Hard-civic	0.8	8.4	–	–
4. Semi-civic	0.2	2.4	–	–

*hec, hectares*.

Within this communal space provision, Castle residents are furnished with a green space and a play and informal sports area. Whereas Accordia provides two key additional types of COSP: hard-civic and semi-civic. Further details of different types and sub-types of COSP are provided within Sections 1.1 and 2.1 of Supplementary Material. With the exception of the semi-civic space, all COSP within both areas are accessible to the general public, as well as local residents.

Accordia residents moved into the first phase of this development in 2006, which is situated on the southern edge of Cambridge city center, in the UK. Castle is found on the northern edge of the same center and was built largely in the twentieth Century. Both neighborhoods are well-educated, middle-to-high-income areas, which are fairly typical of Cambridge but not representative of the wider UK (28). Prior to empirical study, 2011 UK Office of National Statistics (ONS) data were collated at Output Area (OA) and Lower Layer Super Output Area (LSOA) levels. These showed residents in Accordia and Castle have, on average, very similar levels of education, home ownership, household composition, employment status, self-reported health, and constitute largely of a white ethnic background (28). As well as having similar socio-demographics, the two neighborhood were matched in terms of general urban design characteristics. For example, they share similar levels of arterial road connectivity, building density, massing, building line, and land-use. Both areas are relatively disconnected from their immediate hinterland, but Accordia is moderately more cut-off and cul-de-sac in nature. Further details of the matching are provided within Sections 1.2 and 1.3 of Supplementary Material.

### Participant selection

A postal survey was distributed to each household within both neighborhoods. The questionnaire was presented with a cover letter and an incentive prize draw, providing the possibility of winning generic shopping vouchers. Approximately 32% of the households in each neighborhood completed the postal questionnaire. The second mode of participant data collection was direct behavior observation. This involved observing residents going about their lives within each neighborhood’s COSP. Observations were not limited to particular socio-demographic grouping. All questionnaires and behavior observations were carried out between April 22nd and June 13th, 2012. The questionnaires were administered at the beginning of this period and observations were gathered at consistent intervals throughout the 8-week period. No follow-up measures have been undertaken to date.

### Variables

As outlined above, the key outcomes studied were the Five-Ways health behaviors closely associated with sustained well-being, each of which were included as *a priori* variables within the questionnaire. Additionally, the pursuit of the Five-Ways generally and locally were distinguished, with the latter forming the principle focus of enquiry. Behavior observation of local residents focused on the first three of the Five-Ways, since they are particularly relevant to urban design, namely, Connect, Keep Active, and Take Notice (i.e., the Three-Ways). The remaining two, “keep learning” and “give” are less easy to observe and measure in the context of people using outdoor space. Accordingly, the following three behavior types were examined:

1. Connecting with other people, whether familiar or strangers (e.g., talking and listening);
2. Engaging in physical activity (e.g., competitive or casual ball games) and;
3. Taking notice or being aware of one’s external environment (e.g., watching wildlife).

The primary predictor variable consisted of the proportionate amount of COSP, in relation to private outdoor space provision (POSP). Volume of COSP refers to the amount of space within a neighborhood boundary that is not inhabited by residential, commercial or public buildings, or private gardens. Whereas POSP typically excludes private building footprints that includes private garden areas and driveways. Together, these can be expressed as a simple ratio of COSP:POSP. The secondary predictor of health behaviors in this study was type of COSP. This paper concentrates on four key categories: green space, play and sports, hard-civic, and semi-civic. Two key sub-types of hard-civic space are also highlighted: *home zone* and hard-landscaped spaces. Each of the above categories and sub-categories are described in more detail within Supplementary Material.

Potential confounding factors were considered within the study. In particular, socio-demographics that may be correlated with health behaviors were taken into account. For example, level of education, which may theoretically be associated with continued learning through life. Care was taken to check whether these potential confounding characteristics were found to be significantly different between the two areas.

Finally, selection and detection biases were also incorporated. Selective migration is a critical factor when considering the potential causal role of COSP:POSP ratio (3, 4). This bias involves residents moving to an area that suits their needs or predispositions, rather than moving to an area which, in turn, influences their health behaviors. Two types of detection bias were also incorporated: (a) population size and (b) *visiting* users. First, the two neighborhoods are built at similar housing density but one has a larger geographical area and therefore includes more housing. The two neighborhoods also varied in average persons per household. The combined differences in the number of houses and household size affect the likelihood of observing residents outdoors (i.e., higher critical mass brings greater likelihood of direct observation). The two factors were united as an estimated population size variable to be incorporated in behavior observation analyses. Second, in order to address the principle research question within the behavior dataset, the proportion of non-residents was also estimated. The use of this variable ensured that analyses were focused upon the local population and not visitors.

### Measurement

It was not possible to find a single extant robust questionnaire covering the Five-Ways. Instead, a robust bespoke questionnaire was developed. Items were drawn upon from related questionnaires, or new items were created to reflect the five *a priori* constructs. The majority of items within each of the five scales tapped general behaviors that were deemed to serve as a means to attain goals implied by each of the Five-Ways (e.g., “use stairs instead of lifts” – to Keep Active). In order to test the principle hypothesis, several items within each scale included local or neighborhood-specific items (e.g., “play ball games around the neighborhood” – also to Keep Active, but locally). Where new survey items were generated, this was in part informed by focus groups with residents from both neighborhoods. The questionnaire was critically evaluated by experts in metric and scale development and piloted with a small number of *lay* persons.

The volumes of COSP and POSP were measured in AutoCad software, using base plans acquired via Digimap, a service providing maps and geospatial data for higher education in the UK. These maps are authored and licensed by Ordnance Survey. Types of COSP were determined using two separate taxonomies. The first represents previous government planning guidance PPG17: Planning for Open Space, Sport, and Recreation (29), which includes criterion for distinguishing nine types of space. The second framework authored by Danish urban designers and architects (30) offers 12 types of space, several of which overlap with PPG17 categories. The two frameworks were selected as together they represent a comprehensive coverage of both hard urban space types (e.g., squares and boulevards) and green space types (e.g., allotments and parks). Four additional sub-types of space were also introduced where neither PPG17 nor Gehl categories offered sufficient description (see Supplementary Material). Assessment criteria were applied by an experienced urban design practitioner (Jamie Anderson) and checked by a qualified architect and professor of environmental design.

A customized instrument was also created for the direct observation of the Three-Ways. Activity codes were chosen to reflect the Three-Ways behavior types, representing a combination of *nef* example behaviors, System for Observing Play and Recreation in Communities (SOPARC) coding tool (31), together with preliminary observation carried out during pilot stages. A clear implementation protocol was also piloted drawing upon the validated SOPARC tool. Implementation involved the researcher blending into COSP settings. For example, observation sessions were undertaken on a bicycle in the public highway and activities were coded, once residents had been passed. A digital tablet with a 3G connection was utilized, giving the impression of someone checking e-mails or surfing the Web. Where possible, a primary and secondary activity, as well as subjects’ gender, estimated age and location within the setting were coded, using a predefined list (Supplementary Material).

Each neighborhood was observed five-times-a-day, five-days-a-week including weekends, Fridays (a unique weekday) and two other random weekdays. This selection is based on the work of Hardie et al. (32) who found these days to be representative of a typical week. In order to avoid normal weekday working and school times, observation sweeps were made at approximately 8 a.m., 12.30 p.m., 4 p.m., 5.30 p.m. and 7 p.m. A coin was tossed to decide which neighborhood to visit first and observation sessions, conducted in the separate areas, were made within 20 min of each other. The use of a coin introduced randomization to the collection process and the close-knit timings helped to ensure that weather conditions were similar for both areas, in each session.

Ethical approval was sought for a separate strand of the study involving participants wearing global positioning system (GPS) recorders but, not for direct behavior observation. This aspect of the research did not involve the following of individuals or reveal personal information. Instead, a community was observed going about day-to-day life in public highways and outdoor neighborhood spaces. Anonymity was maintained within the mapped representation of data.

In order to understand the main reasons why residents chose to live in their neighborhood, an instrument developed by Frank et al. (33), was incorporated to the questionnaire. The instrument includes 12 items (i.e., 12 reasons). Three new reasons were added that had featured within focus groups in both neighborhoods. These included (1) Quality of architecture; (2) Proximity to communal green; and (3) Immediacy of recreational and cultural opportunities. Participants were required to rank the appropriate level of importance using a five-point Likert-type scale.

Weather data were gathered retrospectively, from the National Climate Information Centre (NCIC) and the proportion of visitors was measured via a survey of COSP users across each neighborhood (*n* = 189). The survey involved the researcher presenting users with *a priori* neighborhood boundary (defined by local residents). Where a participant stated that they live outside this area; for the purposes of the study, they were deemed to represent a visitor. Additional potential cofounding, such as cultural influences (e.g., festivities) and economic factors (e.g., permanent opening or closure of shops), were also monitored throughout the 8-week study.

### Study size

As the research question concentrated on residents living within each of the two neighborhoods, the size of the questionnaire study was limited to all households within these geographic areas and the number of postal surveys returned. The size of the behavior observation study was not based on formal power calculations. Instead, a sufficient number of observation periods were calculated based on pilot observations and conservative estimates of persons expected, per observation session. Pilot work showed that, on average, four to seven people engaged in one of the Three-Ways, per session and area. This was considered to represent a conservative expectation for a typical session in spring. On this basis, it was estimated that 200 observation sessions would provide a minimum of 800 observations in each neighborhood. This projected dataset was deemed large enough to detect small Three-Ways effect sizes, with sufficiently narrow confidence intervals to provide statistically meaningful insights.

### Quantitative variables

Within the design of the Five-Ways questionnaire and subsequent analyses, two key groupings were applied. Where possible, survey items sought to use frequency response options with as much specificity as possible. However, rather than using an artificially precise continuous measure, seven and five-point descriptive scales were used. Where an item sought information on a memorable activity, such as *playing a competitive ball game*, participants were asked how often they had engaged in the behavior in the past 4 weeks using a seven-point descriptive scale, ranging from *never* to *everyday*. In the case of more informal and episodic activity, such as *greeting people around the neighborhood*, a five-point adverb-based scale was used, ranging from *never* to *always*. The second grouping within the survey dataset involved the interpretation of factor analysis (FA), using Varimax and Oblimin rotations. This process involved the use of established objective procedures to find interpretable item groupings, which were named on the basis of face-value interpretation. Further details of both grouping approaches are provided in Section “Specific Factor Differences” and are to be reported elsewhere.

Once coded using specific behavior types, observation data were combined into one of the Three-Way categories. This process was based on the interpretation offered by *nef*, Jamie Anderson, and examples provided by local residents within focus groups. Analysis of participants ranking of reasons for choosing their neighborhood involved using the cut-point “very important.” Where a resident selected this response option, it was considered to be indicative of a strong preference for a local resource or opportunity, at the time of choosing among alternatives.

### Statistical methods

All statistical analyses were undertaken in SPSS software. Categorical socio-demographic information for each neighborhood was compared using Chi-Square tests of independence. Where quantitative scale measures were used, independent *t-tests* were used to compare areas. Statistically significant differences (*p* > 0.05) were not found for potential socio-demographic confounding factors and were therefore not used within the subsequent Five-Ways analysis. The author is not aware of factors that are strongly associated with the Five-Ways and should be controlled when small differences are found.

Factor analysis was utilized to test the independence of Five-Ways factors and reduce the dimensionality of the data, increasing the power of *post hoc* testing of differences between neighborhoods. Varimax rotations were used within the first FA and a number of solutions were explored around a specific number of factors. Items found to consistently cross-load, on at least two or three factors, and/or represent low loaders (<0.4) within each of the factor solutions, were removed to optimize validity. In addition, to safeguard against artifactual creation of facto structure, the pattern of high-loading items was also checked using a non-orthogonal Oblimin solution. Where valid and reliable *a priori* factors were uncovered, differences in area factor scores were tested using independent t-tests. Further details of the FA are to be published elsewhere.

Robust factors were totaled in an optimally weighted way by taking their first principle component (PC) as a way in which to summarize covariance in FA. Specifically, the items that loaded highly on confirmed *a priori* factors were combined into a location-specific score by this means. The weighting in a PC is optimal in the sense of allowing a larger role for items best correlating with all other items considered, which selects mildly for validity and reliability but does not sacrifice reliability by reducing the weighting of related items to 0 (i.e., not discarding them). The same procedure was replicated for the remainder factors, which, on the whole, consisted of generally rather than locally worded items.

Population size was calculated by multiplying the number of houses in each area by the average household size reported by residents within the postal survey. The proportionate difference in population size between the two areas was subtracted from overall aggregated behavior counts, in the larger neighborhood. The estimated proportion of visitors was also calculated for each separate area. This weighting calculation was based on the aforementioned user survey. The estimated proportion of visitors, in both areas, was also subtracted from the overall behavior counts. Once population size had been weighted and visitors extracted, percentages and percentage differences for each of the Three-Ways were then compared between neighborhoods. A measure of association was performed using the Pearson Chi-Square non-parametric test. Care was taken to check that assumptions of the test were not violated. In order to establish whether a result was statistically significant, the resulting Chi-Square score was compared to an appropriate critical value in a Chi-Square distribution. In order to establish magnitude of association a standardized odds ratio (OR) measure was chosen and used to calculate effect size measure was calculated.

The majority of participants in both neighborhoods completed most survey items. On average, 10% of responses were missing from each questionnaire item. Once a feasible model had been achieved using FA, the same model was replicated with the complete dataset and SPSSs imputed *mean* function was selected for missing cases. In the instance that an observation session had been missed within the behavior observation dataset, imputed calculations were not used, as a small proportion were missing (4%) and the available data were sufficient to obtain statistically significant findings.

## Results

### Survey participants and bias

#### Participants

Approximately 320 questionnaires in Accordia and 480 questionnaires in the larger Castle were posted to individual households. Approximately 30% of potential households in each neighborhood completed and returned the survey. The median age of participants was 47 in Accordia and 54 in Castle. Although age range in both areas was relatively wide, children and teenagers below the age of 17 did not complete the questionnaire and are therefore not represented within the survey findings.

The cut-off date for the use of returned survey data were 8 weeks, coinciding with the completion of the behavior observation period. After 4 weeks, it was noted that substantially more females had responded than males. A request for more surveys completed by males was circulated via local Resident Associations. The number of male respondents increased but, overall, around 10% more females responded in both areas. No further attempts were made to ensure that responses were representative. Where residents participated, it was commented that the questionnaire was interesting and well-presented. Reasons why the survey was not completed and returned by the majority of households were not uncovered.

Table 2 shows that most socio-demographics were not found to be statistically different between the neighborhoods. Six factors exhibited statistical differences, including married persons, single person households, size of average household, households with dependent children, household income, and average length of residency. The household size and number of dependent children differences may be explained by Accordia’s average physical house size that, on average, is larger and lends itself to the accommodation of families. Castle also has a marginally higher proportion of lodgings rented by students whom listed themselves as single person households.

**Table 2:** **Summary comparison of socio-demographics in Accordia and Castle**.

	Accordia	Castle	χ*^2^* (*df*)	t (*df*)
Women (%)	58	61	0.21 (1)	
White ethnic background (%)	86	95	5.89 (1)	
Divorced, separated or widowed (%)	12	17	1.53 (1)	
Married (%)	74	58	6.23 (1)*	
Single person households (%)	14	24	6.28 (1)*	
Average household size (persons)	2.9 (1.4)	2.2 (1.1)		3.9 (178)***
Households with dependent child(ren) (%)	50	27	13.45 (1)***	
Household income > £80k (%)	42	19	17 (1)***	
Average residence (years, SD)	4 (6.1)	14 (13.5)		−8.1 (230)***
Age (median)	47	54		
Average full-time education (years, SD)	17 (3.7)	17 (3.8)		0.83 (214)
Employed or in full-time education (%)	75	81	1.63 (1)	
Self-reported good health (%)	89	89	0.45 (2)	
Activity restricted by disability (%)	5	9	0.18 (1)	

*p < 0.05.

***p < 0.001.

Household income and the average residency showed among the most significant differences (*p* < 0.001) between the neighborhoods. The latter difference is expected as Accordia’s first phase of development was completed just four years before the questionnaire was distributed. Whereas the Castle area was built earlier and experiences a stable population, exceeding the 10-year average tenure in England (34). A household income of above £80,000 per annum was selected as an appropriate level of comparison because the proportion of people above this threshold, in both areas, represents the most substantial difference. For instance, in Accordia 42% of participants reported a household income above £80,000, compared to 19% in Castle. This difference may, in part, be explained by a higher proportion of single person households in Castle.

Of the six items that exhibited significant differences, it is possible that household income and average residence may confound the local Connect and Give outcomes. It is possible that in Castle, where the average residency is 10 years longer than in Accordia, residents may have accrued a stronger breadth and depth of local social relations, interacting more often with their neighbors.

#### Neighborhood Selection

Participants were asked to indicate the most important reasons why they chose to live in their neighborhood. The percentage of participants who ranked each reason as “very important” is shown for each neighborhood, in Table 3. It can be seen that most items were not found to be statistically different *(p* > 0.05). The seven items in the top half of the table were all found to represent statistically significant differences.

**Table 3:** **Reasons for choosing neighborhood that participants strongly agreed with (%)**.

Reason	Accordia	Castle	χ^2^ (*df*)
Tranquility of area or street (%)	4	42	43.2 (1)***
Access to private garden (%)	26	57	19.26 (1)***
Quality of architecture (%)	46	19	20.53 (1)***
Quality of schools (%)	52	24	16.1 (1)***
Near public transport (%)	46	24	12.71 (1)***
Affordable housing (%)	18	37	8.25 (1)**
**Near communal green spaces** (%)	42	27	5.61 (1)**
**Ease of walking** (%)	57	48	–
Low crime (%)	45	35	–
Access to major roads (%)	21	14	–
Close to place of work (%)	33	39	–
**Close to cultural opportunities** (%)	33	29	–
Near shops and services (%)	30	27	–
**Close to sports facilities** (%)	6	2	–
**Sense of community** (%)	23	22	–

**p < 0.01.

***p < 0.001.

The five items highlighted in bold represent potential confounding factors, as they demonstrate value participants assigned to resources associated with their ability to Connect, Keep Active, and Keep Learning (i.e., being *close to sports facilities* would facilitate Keep Active behaviors). Therefore, a strong preference is taken as an indication of participants’ predisposition for the corresponding health behavior. It is shown that four of these items were statistically similar in both areas, demonstrating that for three of the Five-Ways (Keep Active, Connect, and Keep Learning), the two areas are well matched and do not exhibit bias within these domains. It follows therefore that, for the survey dataset, it is unlikely that differences detected between the two neighborhoods are due to differences in residents’ general attitudes. Evidence to suggest that these factors are strongly linked with the outcome variables was not found and non-significant differences were not controlled.

The fifth bold item (*near communal green spaces*) shows a statistical significant difference between areas (*p* < 0.01). Given that statistical differences were not discovered for the “ease of walking,” “close to sports facilities,” and “sense of community” items, it is less likely that Accordia residents’ stronger preference for communal green space, compared to Castle, is indicative of this group generally being more physically active or sociable. It is not clear whether Accordia residents may be more *mindful* (i.e., predisposed to Take Notice cognitions). Key factors listed by a small proportion of respondents, under *any other reasons* (i.e., not covered in the questionnaire) included being near to family and limited choice.

### Main survey results

#### Factor Analysis

It was not possible to find a single extant robust questionnaire covering the area of the Five-Ways. Instead, a bespoke instrument was compiled to reflect the five *a priori* constructs. FA was used to reduce 56 items to a compact set of dimensions and to assess the empirical adequacy of the general and local Five-Ways *a priori* concepts (i.e., 2 × 5 = 10 in total). A list of 44 retained items is provided within Section 1.5 of Supplementary Material.

Seven meaningful factors were extracted and named on the basis of face-value interpretation of high-loading items:

1. Attitude to Neighborhood Life
2. General Attitude to Nature
3. General Attitude to Close Personal Relations
4. Attitude to COSP Usage
5. General Attitude to Giving and Sociability
6. General Attitude to Keep Learning
7. General Attitude to Keep Active.

It can be seen that of the 10 *a priori* constructs planned for evaluation, just 2 (Keep Learning and Keep Active) materialized as factors with sufficient construct validity. This meant that 8 of 10 intended specific hypotheses (Five-Ways locally and generally) could not be tested according to psychometrically conventional ways of defining measures. However, it was still possible to test hypotheses for the *a priori* constructs, as well as other hypotheses that are useful to the research objectives of the study.

#### Neighborhood Differences

**Figure 1:**
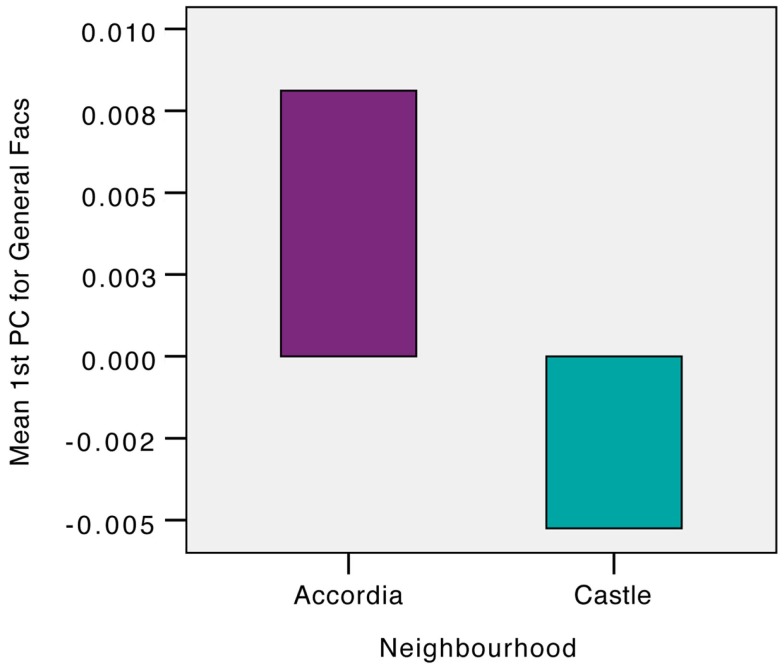
**Comparison of average first PC scores on general factors for Accordia and Castle**.

**Figure 2:**
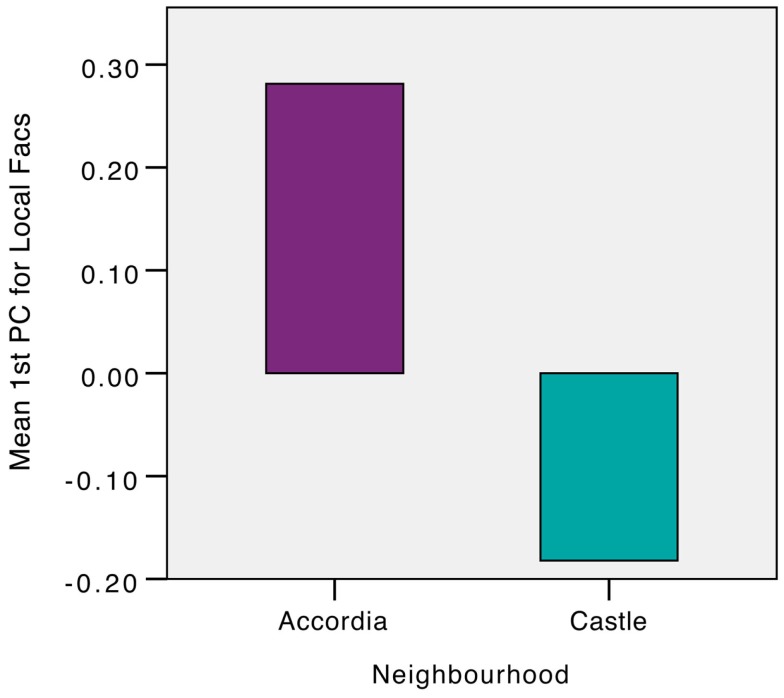
**Comparison of average first PC scores on local factors for Accordia and Castle**.

The items that loaded highly on Factors 1 and 4, the two apparently location-specific factors, were combined into a location-specific score. This was repeated for the remainder factors that comprised largely of generally rather than locally worded items. Figures 1 and 2 compare the average general and local PC scores for the two neighborhoods. Independent *t*-tests were then undertaken to examine average differences between the neighborhoods on general and local PC scoring.

#### General Factor Divergences

On average the general first PC score (i.e., of items not in the location-specific factors) appeared to be slightly higher in Accordia (*M* = 0.008, SE = −0.09) compared to that observed in Castle (*M* = 0.005, SE = 0.08). This difference was not significant *t*(255) = 0.11, *p* > 0.05 and had a very small effect *r* = 0.02.

#### Local Factor Divergences

On average the local first PC score was found to be higher in Accordia (*M* = 0.28, SE = 0.09) compared to that observed in Castle (*M* = −0.18, SE = 0.08). This difference was found to be significant *t*(255) = 3.72, *p* < 0.0001 and represented a substantial medium sized effect *r* = 0.23.

Together, these results show that, in general, the two groups do not differ in their general dispositions in the domain of community as measured by the items loading on the majority of factors extracted via FA. However, in spite of these resemblances, the Accordia residents reported that they were much more likely to pursue neighborhood-specific activities.

#### Specific Factor Differences

**Figure 3:**
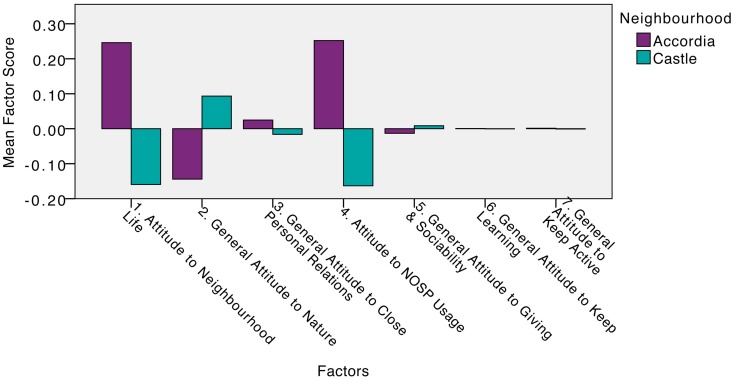
**Comparison of seven average factor scores, for Accordia and Castle**.

In order to explore whether the dataset might allow any further conclusions beyond the explicit *a priori* hypothesis related to Keep Learning and Keep Active, as well as establishing whether the residents in Accordia and Castle exhibit additional specific differences, a MANOVA was performed with all seven factor scores. Figure 3 compares mean factor scores for the two neighborhoods.

Sample sizes in the two neighborhoods were not equal and Box’s test was not significant (>0.05), providing assurance that the assumption of equality of covariance was not seriously in error. Using Pillai’s trace, a significant overall difference was found between the Accordia and Castle groups for the seven factor scores, *V*  = 0.09, *F*(7, 249) = 3.72, *p* < 0.001.

Simple univariate contrasts were then used to compare the two neighborhoods, for the factor scores revealed on each of the seven factors. The following summarizes the differences for each factor, with one and four repeating in parts what was already shown in the combined hypothesis test:

1. *Attitude to Neighborhood Life*. Accordia was found to be significantly higher than Castle for the locally specific sociable activities, *F*(1) = 10.45, *p* < 0.001.
2. *Attitude to Nature*. No significant difference was discovered between the neighborhoods for items pertaining to positive behaviors associated with nature, *F*(1) = 3.48, *p* > 0.05.
3. *Close Personal Relations*. No significant difference was uncovered between the neighborhoods for general close relations, *F*(1) = 0.10, *p* > 0.05.
4. *COSP Usage*. Accordia was found to be significantly higher than Castle for local residents’ usage of outdoor neighborhood spaces, *F*(1) = 10.57, *p* < 0.001.
5. *Giving and Sociability*. No significant difference was found between the neighborhoods for combined general giving and sociability items, *F*(1) = 0.03, *p* > 0.05.
6. *Keep Learning*. No significant disparity was discovered between the neighborhoods for general continued learning behaviors, *F*(1) = 0.00, *p* > 0.05.
7. *Keep Active*. No significant difference between the neighborhoods was found for typical Keep Active behaviors, *F*(1) = 0.00, *p* > 0.05.

With the possible minor exception of Factor 2, all the factors used in this exploration produce strikingly small differences. The Keep Learning and Keep Active similarities strengthen the neighborhood selection findings outlined earlier. Both show that the residents of the two neighborhoods had similar reported general attitudes, in most respects tapped by the questionnaire. It is therefore improbable that any difference between neighborhoods in respect of these two specific dispositions would confound specific local insights revealed.

The Attitude to Neighborhood life score was significantly higher within Accordia than in Castle. This measure is made up of the largely pro-social behavior items (see Supplementary Material), which are specific to neighbors and the local vicinity. Moving to consider a more specific form of control against confounding, the Close Personal Relations factor also consists of pro-social activity, but of a type that is more general; this was very similar between the neighborhoods. It is therefore not likely that the more frequent pursuit of neighborhood behaviors within the Accordia survey sample arises because of any greater friendliness or sociability of the people.

The Attitude to Nature, Giving, and Sociability factors do not correspond closely enough to the Five-Ways *a priori* concepts to warrant specific comparison.

### Behavior observation sample

A total of 191 observation sweeps were made on a bicycle, to both Accordia and Castle. A total of 382 neighborhood sweeps were made (approximately 65 h of observation). Altogether, 3,758 people were observed in Accordia and 3,540 in Castle.

#### Gender

The proportion of women observed was higher than men in both areas (58% in Castle and 56% in Accordia). A marginally higher proportion (2%) of females was observed than males in Castle, compared to Accordia.

#### Estimated Age

**Figure 4:**
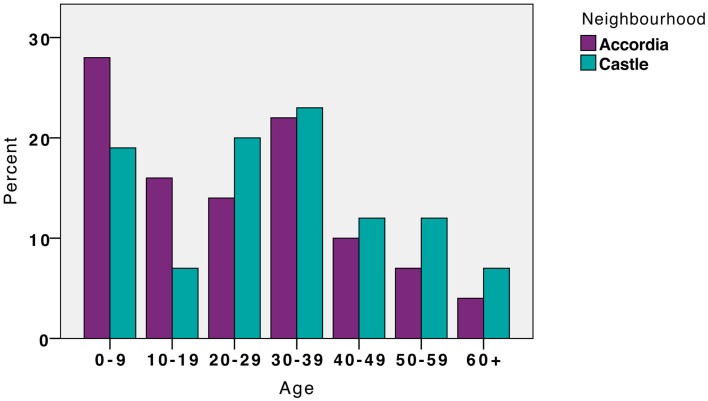
**Estimated age groups of observed subjects (%), in Accordia and Castle**.

Figure 4 unpacks the age profile of the two observed groups. This chart shows that the ages were similar in each neighborhood but a higher proportion of those people observed in Accordia were children or teenagers. In Accordia, 28% of those people observed were nine years old or younger, compared to 19% in Castle. Also, in Accordia, 16% were estimated as being between the ages of 10 and 19, contrasted with 7% in Castle. In contrast, Castle exhibited a higher percentage within all the age brackets above the age of 19.

### Main observation results

#### Observed Three-Ways Activity

Section 1.4 within Supplementary Material provides a summary of all aggregated primary and secondary Three-Ways behaviors observed, once differences in population size and visitors were taken into consideration (i.e., the proportionate difference was subtracted). Non-Three-Ways activities were also recorded and are listed within this table. The results outlined here concentrate on users deemed to be engaged in one of Three-Ways. Counts were aggregated into socializing (Connect), being physically active (Keep Active) and showing an interest in the immediate social or physical environment (Take Notice) categories, for each neighborhood.

Accordia was associated with both significantly (*p* < 0.001) and substantially more healthy behaviors, summarized in the fourth column of Table 4. Between Accordia and Castle, there was 31% difference in Connecting, 29% more Keeping Active, and 4% more Taking Notice. ORs showed that it was 3.58 times more likely to observe Connect activities in Accordia than in Castle. This finding concurs with the self-reported Attitude to Neighborhood Life reported above. It was 3.38 and 3.58 times more likely to see people Keeping Active and Taking Notice in Accordia, respectively. However, as described in Section “Factor Analysis,” coherent local factors for these *a priori* constructs did not emerge during FA. Therefore, direct comparison with the self-reported activities was not possible.

**Table 4:** **The number of Three-Ways observed, per cent difference and magnitude of effect**.

Outcome	Accordia count	Castle count	% Diff.	Effect size (OR)
Connect	2,579	1,141	31*	3.58 (medium)
Keep active	2,587	1,451	29*	3.38 (medium)
Take notice	225	52	4*	3.58 (medium)

*p < 0.001.

#### Where the Activities Occurred

**Figure 5:**
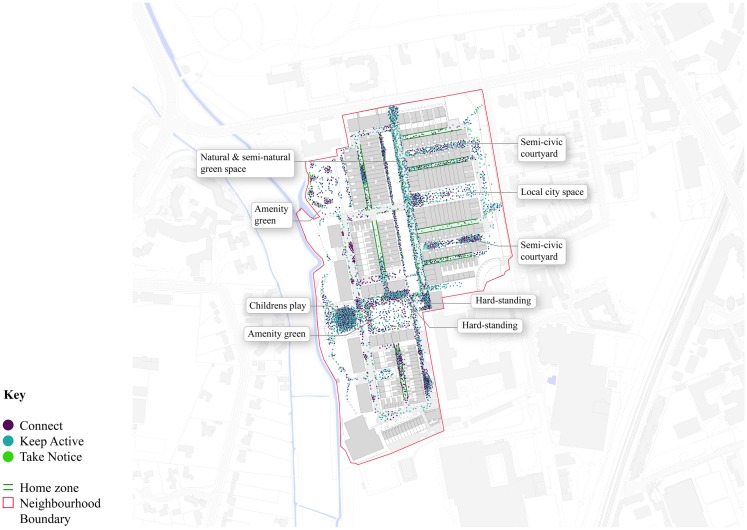
**Location of the Three-Ways activities observed in Accordia**.

**Figure 6:**
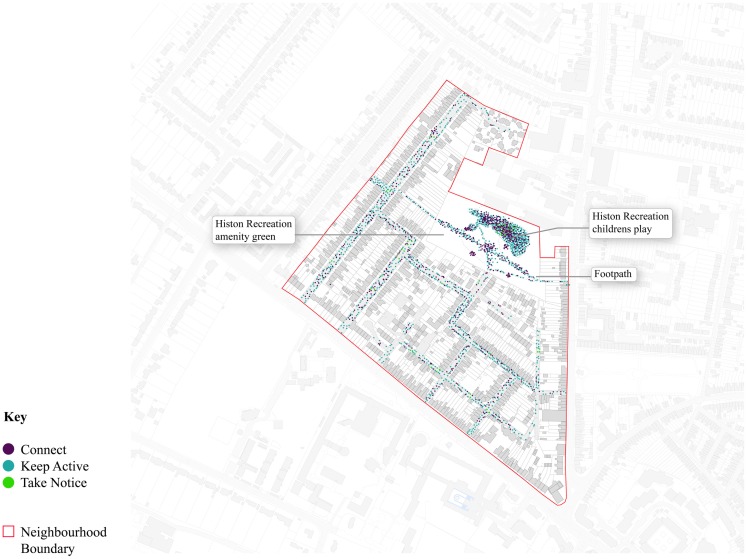
**Location of the Three-Ways activities observed in Castle**.

In order to answer which types of COSP were associated with the Three-Ways activity, the location of behaviors were mapped and analyzed. Each dot in Figures 5 and 6 is placed in the approximate location coded by the researcher at the time the sighting was made. Unlike the observation analyses outline above, the dots represent observations of local residents and visitors.

Figures 5 and 6 exhibit two clear patterns of Three-Ways behavior that are very similar. Most distinctively, in both Accordia and Castle, high levels of clustering of Connect and Keep Active behaviors can be seen in the children’s play areas. In Castle, the precise outline of the play area is deciphered by these purple and teal dots. In Accordia, the main children’s play area can also been clearly identified by high clustering to the west of the study boundary. In both areas, the children play areas are the most used type of COSP within each neighborhood. Although more difficult to distinguish (light green dots), both neighborhoods have a high proportion of Take Notice activity concentrated within their play areas.

These figures show that the largest spatial differences in behavior were associated with the streets. For example, in Figure 5 the purple and teal dots show the number of persons who Connected and Kept Active in the Accordia home zones (a hard-civic COSP sub-type) and local street network. This is compared to Figure 6, which shows considerably fewer purple and teal dots in the Castle local streets. Fewer activities can be observed along the pavements in Accordia and the majority of activity in Castle’s streets represents cyclists passing through, instead of subjects (particularly children and teenagers) lingering to play, or talk among themselves.

Sporadic indications of Keep Active, Connect, and Take Notice activity were observed on the grass areas in both neighborhoods. Within Accordia (Figure 5) relatively few dots can be observed in the green spaces, compared to the hard-civic spaces (e.g., local city space and hard-standing) semi-civic areas, providing indication of the popularity of these hard spaces among users.

#### Weather

**Figure 7:**
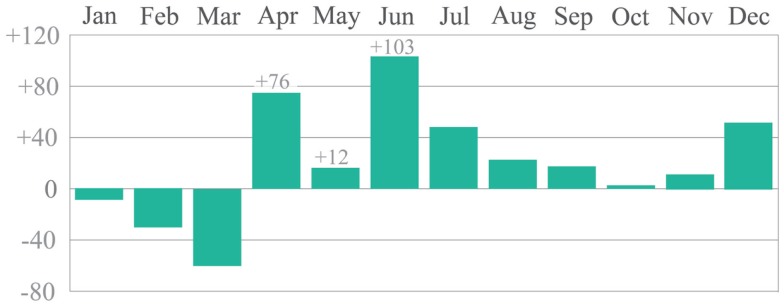
**Percentage difference from monthly average rainfall in 2012 (Source: NCIC (35))**.

All questionnaires and behavior observations were completed during an 8-week period between the end of April and the beginning of June 2012. Traditionally, Cambridge is in the driest region of Britain (35). However, during the 9-week study period, the NCIC, the UK’s official climate center recorded the wettest April and June on record (Figure 7). May also saw above average rainfall, which was typical of the UK’s second wettest year, according to national records starting in 1910 (36). Much of the rain experienced during the study period was extremely heavy, with several bouts of hail and thunder (35). Temperatures were erratic, with some days around or above average, and one recording of ground frost was made in June.

It is likely that the highly wet, cool and sometimes windy weather had a pronounced negative impact on the amount of activity observed within the neighborhood spaces, as found in the context of these conditions elsewhere (37). This observation is reinforced by the researcher’s comparison to a pilot study undertaken within the same neighborhoods in 2011, approximately a year earlier.

#### Social and Cultural Confounders

Additional confounding factors were also considered. Throughout the study period and at intervening periods before and after the study, instances of cultural events, the opening or closure of businesses and social influences, such as crime – were monitored via local media and conversation with residents. Both neighborhoods celebrated Queen Elizabeth II’s Jubilee during the study period and, shortly before the start of the 9 weeks, Accordia benefited from the introduction of a small Londis franchise grocery outlet. The opening of this shop helped to improve matching with Castle, which has two equivalent shops on the edge of this neighborhood. In sum, evidence of potential social and cultural confounding factors was not found. It is therefore unlikely that the study findings have been unduly influenced by variation within these factors in either of the two areas.

## Discussion

### Key findings

It was hypothesized that Accordia’s COSP would be associated with higher levels of (a) resident’s self-reported pursuit of each of the Five-Ways locally and (b) Accordia residents’ observed engagement in each of the Three-Ways, within local COSP. The former (a) was confirmed among adults (17 and over) for Connect and Give activities via Attitude to Neighborhood Life and partially established for Keep Active behaviors, via the COSP Usage factors. FA results did not permit the testing of area differences for Take Notice and Keep Learning *a priori* factors. The latter hypotheses (b) were verified for each of the Three-Ways (Connect, Keep Active, and Take Notice) among adults, teenagers, and children. It is inferred that the behavior findings support the hypotheses confirmed within the self-reported dataset for adults over the age of 17. The observation findings also show that pursuit of the Three-Ways among children and teenagers was higher in Accordia COSP, compared to Castle COSP.

Behavior mapping revealed specific spatial patterns of association. Compared to Castle, considerable amounts of Three-Ways activity in Accordia were found within the local streets (most of which were home zones). Within Accordia, user activity was more strongly apparent in additional types of civic-hard space (e.g., hard-standing and local city space) and the semi-civic spaces, when compared to the green spaces. The survey also shows that the study findings were unlikely to be confounded by differences in socio-demographics, general attitudes to the Five-Ways, or selection bias.

### Comparison with previous research

To the best of the author’s knowledge, this research has not been conducted with measures of Take Notice behavior within the same context. Therefore, linking this outcome with previous research is not permissible.

Comparisons can be made for Keep Active, Connect, and Give relations to outdoor neighborhood space design. The case study findings replicate research suggesting proximity, size, and quality of local outdoor amenities are associated with overall physical activity (3, 4, 37, 38). However, the majority of these studies did not include psychometrically validated survey measures and/or objective measures, such as direct behavior observation. While several studies have linked child activity with green spaces (39), this study replicates the finding that home zones are associated with young person activity (40, 41). A handful of behavior observation studies have also linked physical (42) and social activity (43, 44) with specific street characteristics, such as shop frontages and street furniture. The evidence base for Connect and Give show positive cross-sectional relations between self-reported measures and green space (45, 46). Examples of research which examine the role of COSP:POSP ratio are infrequent (5).

Survey findings from this study that are not reported here, show that Accordia was associated with the same prevalence of enduring SWB as Castle (paper in preparation). It is inferred that, on average, Accordia residents’ well-being may not be undermined by the absence of private garden space. Dunnet and Quasim’s Sheffield (UK) study (7) found that among several reported reasons, well-being benefits were thought to be linked in particular to relaxation and the active creation of a pleasant environment. Early research pertaining to a systematic review provides indication that communal garden space can cater for several of the same benefits (9). However, care may be needed to avoid negative impacts (47).

### Limitations

Adverse weather conditions are likely to have limited how representative the findings are for this time of year. However, given expected patterns of climate change, which include greater fluctuation of weather (48, 49), these results may have increasing relevance for the twenty-first century in the UK.

The general applicability of the findings to wider UK society is limited to upper-middle-class populations. The findings are not necessarily generalizable beyond similar groups, who do not pursue cognizant lifestyles. Although a strong response rate was achieved, it is not clear how representative survey participants are of the two residential areas. For example, by chance, people who completed the survey may have a higher predisposition for socialsing – compared to the two-thirds of the neighborhood who did not. The data therefore may not reflect a fair cross-section of either area. Although participants were found to have similar predispositions for social and physical activity, the Frank et al. instrument used to measure neighborhood selection requires psychometric substantiation. When compared to gender ratio in the UK, it was found that males were moderately under represented within the behavior observations and questionnaire findings (28). Within the behavior observation dataset, it is also not clear how closely these subjects are representative of overall neighborhood makeup.

Reliability and validity of insight have been achieved through FA and the comparison of what people report as well as what they are witnessed doing. However, despite drawing upon an established tool (31), the precision of behavior results is not clear without future testing (e.g., inter-rater reliability). It is possible that the higher household income in Accordia may confound the Keep Active results. However, given that both neighborhoods fall within the highest percentile categories of UK household income and differences in physical activity between these groups is small (50), it is likely that any confounding is weak. Finally, although care is taken within this cross-sectional study to account for bias and confounding, findings linking COSP:POSP ratio and types of COSP with well-being behaviors can not be inferred as causal.

### Conclusion and recommendations

The higher proportion of COSP than POSP in Accordia was found to be strongly associated with three forms of behavior related to health and well-being. Accordia provides 36 and 6 m^2^ of communal and private outdoor space per resident, respectively. By comparison, Castle provides 56 m^2^ private and 14 m^2^ communal space per resident. Should the UK Government’s planned garden cities adhere to Accordia as a model, and assuming the same provision standards, a 15,000 home development would require 145 hectares of land, contrasted with 242 hectares if built in the same manor as Castle. In other words, each prospective *Accordia garden city* would take a 40% smaller bite from the surrounding countryside or green belt (calculations provided within Supplementary Material). This would mean that a substantial amount of farmland, natural habitat, and amenity space is salvaged from urban sprawl. Where applied in urban settings, this mode of development would mean that *brownfield* land is used 40% more efficiently. The use of home zones and high-quality hard spaces may also facilitate well-being, particularly among children and teenagers and warrants further investigation.

Should behavioral findings outlined here be replicated elsewhere with experimental research, the Accordia model may be built to promote higher SWB by supporting and/or prompting behaviors closely associated with well-being. In addition, it follows that the Accordia “communal garden” approach, whereby overall quantity of outdoor space provision is relatively low, will require fewer roads to serve smaller neighborhoods. In turn, this model brings potential benefits to ecological sustainability, such as reduced vehicular mileage, and therefore carbon consumption and CO_2_ emissions, and fewer impermeable hard surfaces that interfere with heavy rain or flooding. High quality of COSP is also likely to positively impact on social sustainability (e.g., cultivating general population health). If applied in practice, long-term economic benefits include mitigating the cost of severe weather impact and lowering National Health Service workload and treatment costs.

The UK Government advises that localities should take city design decisions for themselves, on case-by-case basis (1). However, the Accordia model may not be received with universal popularity. Although interviews found Accordia to be popular with most residents (paper in progress), private gardens have been found to be preferable in surveys of the general population (51). Early research provides indication that communal garden space may provide the same benefits as private outdoor space (9). It is also probable that locally accessible communal areas support more social contact among residents than private gardens, in particular, among less mobile groups, such as children, teenagers, older persons, and the disabled. However, it remains to be to be established whether communal space might, on average, exceed private gardens, in terms of magnitude and variety of benefit.

New cities and neighborhoods that include both Accordia and Castle models may prove to be more optimal when local needs and circumstances are considered. Key caveats include the ability to maintain COSP, adequate amounts of household windows overlooking (i.e., providing “natural surveillance”), which may not be afforded by areas dominated by high-rise buildings. It should also be noted that although few Accordia residents have private gardens, these people have the opportunity to seek out solitary outdoor experiences, by taking advantage of an adjacent green corridor that links into surrounding rural areas.

In sum and taken together, previous insights and the findings of this research show that well-designed communal space is strongly associated with well-being. In this study, the benefit is greater than that provided by a comparable neighborhood that has more private garden area than communal space. These findings can be used to help designers, communities, local, and national governments achieve balanced discussion in shaping future development.

## Conflict of Interest Statement

The research represents empirical research undertaken during my Ph.D. Since completing my doctorate, I have undertaken consultancy work for Social Life. I can confirm that Social Life have not paid me to undertake this research or to publish the findings and have not influenced the content of this paper. I declare that all work presented within this paper is my own and that I have no conflicts of interest.

## Supplementary Material

The Supplementary Material for this article can be found online at http://journal.frontiersin.org/article/10.3389/fpubh.2015.00173
