# Supplementary material for: “Living in a Communal Garden” Associated with Well-Being While Reducing Urban Sprawl by 40%: A Mixed-Methods Cross-Sectional Study
Source: Front Public Health. 2015 Jul 20;3:173. doi: 10.3389/fpubh.2015.00173 (PMC4507402; doi:10.3389/fpubh.2015.00173)

frontiers

Supplementary Material

'Living in a communal garden' associated with well-being, whilst reducing urban sprawl by 40%: a mixed methods cross-sectional study

Jamie Anderson[1,2,3]
[1]Cambridge Well-being Institute, University of Cambridge, Cambridge, UK
[2]Social Life (Social Enterprise Company), London, UK
[3]Department of Architecture, Cambridge University, Cambridge, UK
Correspondence: Jamie Anderson

Department of Architecture, 1-5 Scroope Terrace, Cambridge University, Cambridge, CB2 1PX.
anderson.jamie@gmail.com

1. Supplementary tables

1.1 Types of Communal Outdoor Space Provision (COSP)

Table 1 provides a summary of the types of setting identified within Accordia and Castle. Drawing upon previous UK planning guidance (PPG17) and Gehl (2006) criterion, nine types of COSP were used to categorise spaces within and nearby the two neighbourhoods. These categories were chosen as they were found to provide coverage of both green and urban outdoor spaces for most spaces encountered. Although PPG17 has been deleted within UK government guidance, the *primary purpose* categorisation of spaces was found to be useful to the health behavior approach adopted within the study. However, PPG17 was not found to be as useful in the case of urban and hard-surfaced space types.

Table 1 shows eight of a possible nine PPG17 (2005) spaces, and one of a possible thirteen Gehl (2006) types used within the study. Each category was assigned on the basis of the observed primary purpose of the space, as well as key physical and social attributes. A rationale for each designation is provided within the right hand column of this table. Four additional types of space were also introduced to the list of nine. These included a) school/college playing fields; b) hard-standing; c) partial home zone and; d) semi-civic. Although the primary purpose of each of these spaces overlapped with other types, their physical (e.g. natural or hard-landscaped) and social (e.g. public or private) characteristics departed significantly and were therefore considered standalone COSP types. The following provides a brief description of each:

a) School/college playing fields. This space type is very similar to the PPG17 space named 'outdoor sports facilities'. However, the key distinction is that a space, such as a sports pitch, is used solely by students attending the associated educational establishment and not the general public.

b) Hard-standing. This type consists of a simple level area which may vary in size but, is usually concrete and highly suited to *free-wheeling* transport devices used by children and teenagers. The area may not have been designed for these purposes but has been re-appropriated as a space to be physically active and to socialise.

c) Partial home zone. The concept of home zone is legally recognised in the UK. It has been used in reference to residential streets designed to be shared by pedestrians and vehicles, according to principles espoused by Dutch *woonerfen* (Royal Dutch Touring Club, 1977). Accordia includes several streets that adhere to some of these principles but not all. Consequently, this COSP type within Accordia is referred to as a partial home zone.

The home zones include a change in materials from the nearby streets. In Accordia block paving is used to gently vibrate cars as they drive into and through the home zone, to indicate a change in status and encourage slow driving. These spaces are narrower than traditional residential streets, making drivers more aware of their speed relative to immediate physical objects. The space is a vehicular route but walking, standing or playing is encouraged by using a surface without curbs, highlighting a more equal relationship between cars and people across the street. Finally, the home zones provide direct access between homes and the street, with either no front garden or small back yards.

d) Semi-civic. This innovative space is found in Accordia and situated at the rear of family homes (townhouses), in two separate areas. They are relatively long and rectangular in shape, open on two sides and have low fences and small gates at either end. These semi-civic spaces therefore represent a similar concept to a traditional private courtyard but, are looked by the roads at either end. The spaces are furnished with a mixture of resin bound gravel, small pieces of play equipment, raised allotment beds with integrated seating and fruit trees. Although the spaces are privately owned, interview research found that them to be enjoyed (passively) by fellow residents. Children from various parts of the neighbourhood also shared the space with their peers.

In order to simplify results pertaining to types of COSP, the thirteen types were assigned to one of four over-arching categories: 1. Green Space; 2. Play and Sports; 3. Hard-civic and 4. Semi-civic.

Table 1: Summary of 13 types of COSP used within and near Accordia and Castle

	COSP Type	Primary Purpose	Neighbourhood
**Green Space**	1. Natural & semi natural*	Wildlife conservation, biodiversity and environmental education and awareness	Accordia & Caste
	2. Parks & gardens*	Accessible, high quality opportunities for informal recreation and community events	N/A
	3. Amenity*	Opportunities for informal activities close to home or work or enhancement of the appearance of residential or other areas	Accordia & Caste
	4. Green corridors*	Walking, cycling or horse riding, whether for leisure purposes or travel, and opportunities	N/A

		for wildlife migration	
	5. Allotment*	Opportunities for those people who wish to grow their own produce as part of the long-term promotion of sustainability, health and social inclusion	N/A
**Play & Sports**	6. Children's play*	Areas designed primarily for play and social interaction involving children and young people, such as equipped play areas, ball courts, skateboard areas and teenage shelters	Accordia & Caste
	7. Outdoor sports facilities*	Participation in outdoor sports, such as pitch sports, tennis bowls, athletics or countryside and water sports	N/A
	8. School/college playing fields***	Same as outdoor sports facilities but belong to a local school or college who prohibit their use for the general public, most of the time	N/A
**Hard-civic**	9. Cemeteries & churchyards*	Quiet contemplation and burial of the dead, often linked to the promotion of wildlife conservation and biodiversity	N/A
	10. Local city space**	Space that primarily serves local users and incorporates playing (e.g. play equipment and suitable surfaces) and staying (e.g. seating).	Accordia
	11. Hard-standing***	Use of scooters, skateboards, roller-blading, children's bikes. Usually a level plain surface made of concrete.	Accordia
	12. Partial home zone***	Informal play, social and physical activities in residential streets designed to be shared by pedestrians and vehicles.	Accordia
**Semi-civic**	13. Semi-civic courtyard***	Informal play and social activities in a courtyard accessed privately at the rear of homes but is open on two sides and visually accessible to all members of the community.	Accordia

Note: *PPG17 **Gehl ***Novel

1.2 Socio-demographic match

Table 2 provides a breakdown of key descriptive socio-demographic statistics in Accordia and Castle. The data was obtained from the UK Office tor National Statistics (ONS) Output Area (OA) and Lower Layer Super Output Area (LSOA) data for 2011. The OA level insights are representative of the wards in which Castle (Castle Ward) and Accordia (Trumpington) are situated. This information reflects a wider area than that depicted in the priori boundary (as depicted in section 2.1 of supplementary material) and is therefore not entirely representative of both specific areas. The

LSOA boundary is smaller and offers a lower level of discrepancy. It can be seen that each of the statistics are the same or very similar in both areas.

Table 2: Summary of key LSOA and OA socio-demographics in Accordia and Castle

	Level	Accordia	Castle
Education level 4 and above	OA	70%	71%
White ethnic background	OA	68%	73%
Homeowner	OA	47%	47%
No car or van in household	LSOA	32%	31%
Very good general health	LSOA	58%	64%
One person household	LSOA	38%	35%

1.3 Urban design match

Table 3 provides a summary of the non-COSP urban design characteristics of Accordia and Castle. It is shown that most parameters are close in size, or fulfill the same category assignment. The main exception is vehicular access, where Castle has five links and Accordia has just one. However, in both cases, the roads do not pass through either neighbourhood. Each area is therefore severed from their surroundings on two sides, rendering them both to be cul-de-sac in nature and not through routes. A 25% difference in the proportion of blocks that are perimeter blocks exists between the two areas. However, evidence to suggest that this parameter may influence health behaviours was not found and therefore not accounted for within analyses.

Table 3: Non COSP urban design characteristics in Accordia and Castle

Urban Parameter	Castle	Accordia
Distance from city centre (km)	1.5	1.6
Residential density (per hec.)	42	48
Enclosure ratio (ave.)	1:1.25	1:1.50
Perimeter blocks (%)	75	50
No. apartment blocks	7	5
Continuous building line (%)	85	90
Typical house type	Terrace	Terrace
Neighbourhood land use	Residential	Residential

Nearby land use	Retail<br>Residential<br>Light industry<br>Retail<br>Office	Retail<br>Residential<br>Light industry<br>Retail<br>Office
Vehicular road connections (tertiary)	5	1
Connectivity (pedestrian only)	2	1
Average pavement sizes (metres)	1.5	1.5
Average parking ratio (spaces per household)	1:1.25	1:1.3

1.4 Observed behaviour summary

Table 4 depicts an overall summary of all primary and secondary activities observed in each neighbourhood. The table is divided into each of Three-Ways to Well-being and non Three-Ways. In each case, the coding category used in real-time is depicted in the adjacent column (activity sub-type). Each code was assigned to a Three-Ways category on the basis of representing a means to pursue each of the Three-Ways (see section 2.5 in main text). All remainder observations were assigned to the non Three-Ways category.

Table 4: Summary of all behaviour codes and their observation frequency in Accordia and Castle

Three-Ways	Activity Sub-type	Accordia (all users)	Castle (all users*)
Connect	Talking & listening	2,544	1,833
	Phone call, text or e-mails	52	28
	Holding hands, or linked by arm	42	31
	Visiting or knocking on door	77	22
	Sub total	**2,715**	**1,913**
Keep Active	Walking (utilitarian)	406	481
	Walking (strolling)	656	390
	Dog walking	109	114
	Cycling	245	178
	Running	61	28
	Skateboard, rollerblade or scooter	136	8
	Ball game	135	242
	Play (general)	698	145

	Play (equipment)	255	631
	Gardening	22	10
	Sub total	**2,723**	**2,226**
**Take Notice**	Looking around or observing wildlife	226	79
	Photography	8	
	Sub total	**234**	**79**
**Non Three-Ways**	Housework/domestic chores	61	37
	Packing/unpacking car	206	193
	Coming/going from house	58	70
	Headphones	32	19
	Sitting, leaning or standing	723	371
	Eating and/or drinking	12	13
	Reading	10	9
	Smoking	16	14
	Sub total	**1,118**	**725**

*Weighted for difference in estimated population density

1.5 Questionnaire items used for analyses

Table 5 shows the 44 items that loaded on the seven-factor solution that was chosen using Factor Analysis, for the study. Within each factor, items are ordered with the highest loading items at the top and lowest loaders at the bottom of each set. The type of response option corresponding to each item is also shown. Further details of the psychometric analyses and the original list of 56 original items are to be published separately.

Table 5: Items loading for 7-factor solution and used for testing area differences

Factor	Item	Response Option*
Attitude to Neighbourhood Life	I greet people when I am walking around the neighbourhood	Adv.
	I smile at other people when walking in the neighbourhood	Adv.
	I make eye contact with people when walking in the neighbourhood	Adv.
	I make a point of learning neighbours names	Adv.
	I start conversations with neighbours	Adv.
	I take an interest in the lives and backgrounds of my neighbours	Adv.
	I help neighbours e.g. if they are struggling with shopping	Adv.
Attitude to Nature	I make a point of discovering names and the origin of plants and animals	Adv.
	I took an interest in the wildlife in the local area	T/B
	I consciously watched or listened to birds	T/B

	I take notice of the trees, shrubs and flowers around the neighbourhood	Adv.
	I did gardening or tended an allotment	T/B
Close Personal Relations	I contact friends and family on birthdays or special occasions	Adv.
	I show my appreciation to the people who are important in my life	Adv.
	I discuss intimate and personal matters with family or friends	Adv.
	I phoned the people in my life who are important to me	T/B
	I actively contribute to the happiness of friends and family	Adv.
COSP Usage	In summer, I have barbecues or picnics in the neighbourhood	Adv.
	I played informal games around the neighbourhood (e.g. ball games, Frisbee)	T/B
	I sat on the chairs or bench outside a local shop/café and had something to eat or drink	T/B
	I seek out local squares, parks or greenspaces with people in	Adv.
	I roller bladed or went for a jog locally	T/B
	I canoed, swam or punted in or on the River Cam	T/B
Giving and Sociability	I help organise community events such as street parties	Adv.
	I attended organised local community events	T/B
	I volunteered time to maintain or improve the neighbourhood	T/B
	In the past 12 months, how often did you get involved in work for voluntary or charitable organisations?	T/B
	I attended social, music or sports club meetings	T/B
	I attended a book club or attend lectures for pleasure	T/B
Keep Learning	I set learning goals for myself	Adv.
	I love learning new things	Adv.
	I pay attention to how my emotions affect my thoughts and behaviour	Adv.
	I take the chance to try new hobbies or interests	Adv.
	I take time to watch the stars at night	Adv.
Keep Active	I went for a brisk walk	T/B

I walked for pleasure (not as a means of transport)	T/B
I climbed stairs rather than taking lifts	T/B
I appreciated being out in the fresh air	T/B
I participated in Yoga, Pilates or Tai Chi	T/B
I went swimming	T/B
I attended a sports club or gym in my neighbourhood	T/B
I backpacked or mountain climbed	T/B
I participated in martial arts, boxing or wrestling	T/B

Note:
Adv. = 5-point adverb based response option (never, rarely, sometimes, often always).
T/B = 7-point time-based response option e.g. *how often did you do the following in the past four weeks* (never, once in the last 4 weeks, 2 to 3 times in last 4 weeks, once a week, 2 to 3 times per week, most days, every day).

1.6 Area calculations supporting reduced Urban Sprawl

These workings are based on a simple per-capita rate of outdoor space provision. Accordia affords 36m² per resident of Communal Outdoor Space Provision (COSP) and 6m² of Private Outdoor Space Provision (POSP) per resident. Castle provides 14m² of COSP and 56m² per resident of POSP.

Based on ONS (2011) census, an average household size of 2.3 persons per household is assumed in any new 15,000 home development. This would mean a new town or extension of this size would comprise of, on average, 34,500 residents (15,000 x 2.3).

Therefore a new development built with Accordia's provision standards would require 145 hectares of land to accommodate the necessary COSP and POSP for 34,500 people ((36m+6m) x 34,500 = 1,449,000m²). This figure converted into hectares is 145 hectares (/10,000m). Whereas a Castle based development would require 242 hectares of outdoor space provision ((14m+56m) x 34,500= 2,415,000m²/10,000m = 242 hectares).

A development based on Castle provision standards would therefore require 97 more hectares than a development based on Accordia standards (242-145= 97). This was translated into a percentage - 40% more land area for outdoor space provision ((97 x 100)/242).

2. Supplementary figures

2.1. Distribution of COSP

Supplementary Figures 1 and 2 show the distribution of the various COSP types
within and nearby Accordia and Castle, respectively. The red line represents a *priori* boundary that was defined in conjunction with local residents who attended a focus group in each area separately. This exercise involved group interview whereby an initial task was to discuss a redline boundary depicted on an A0 sized printout and included road names/landmarks.

Accordia COSP: Mapping of Types within the neighbourhood & nearby

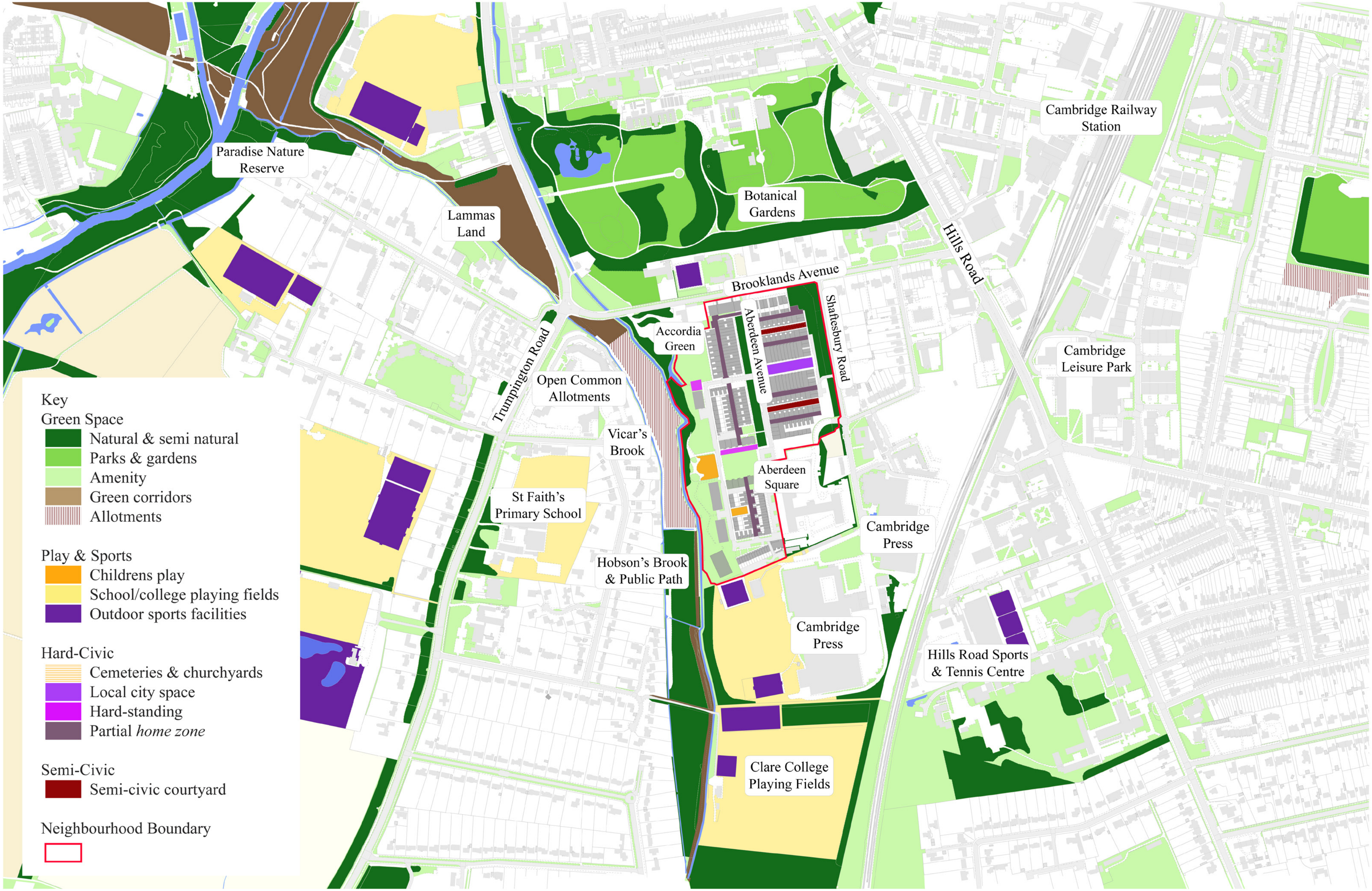

Castle COSP: Mapping of Types within the neighbourhood & nearby

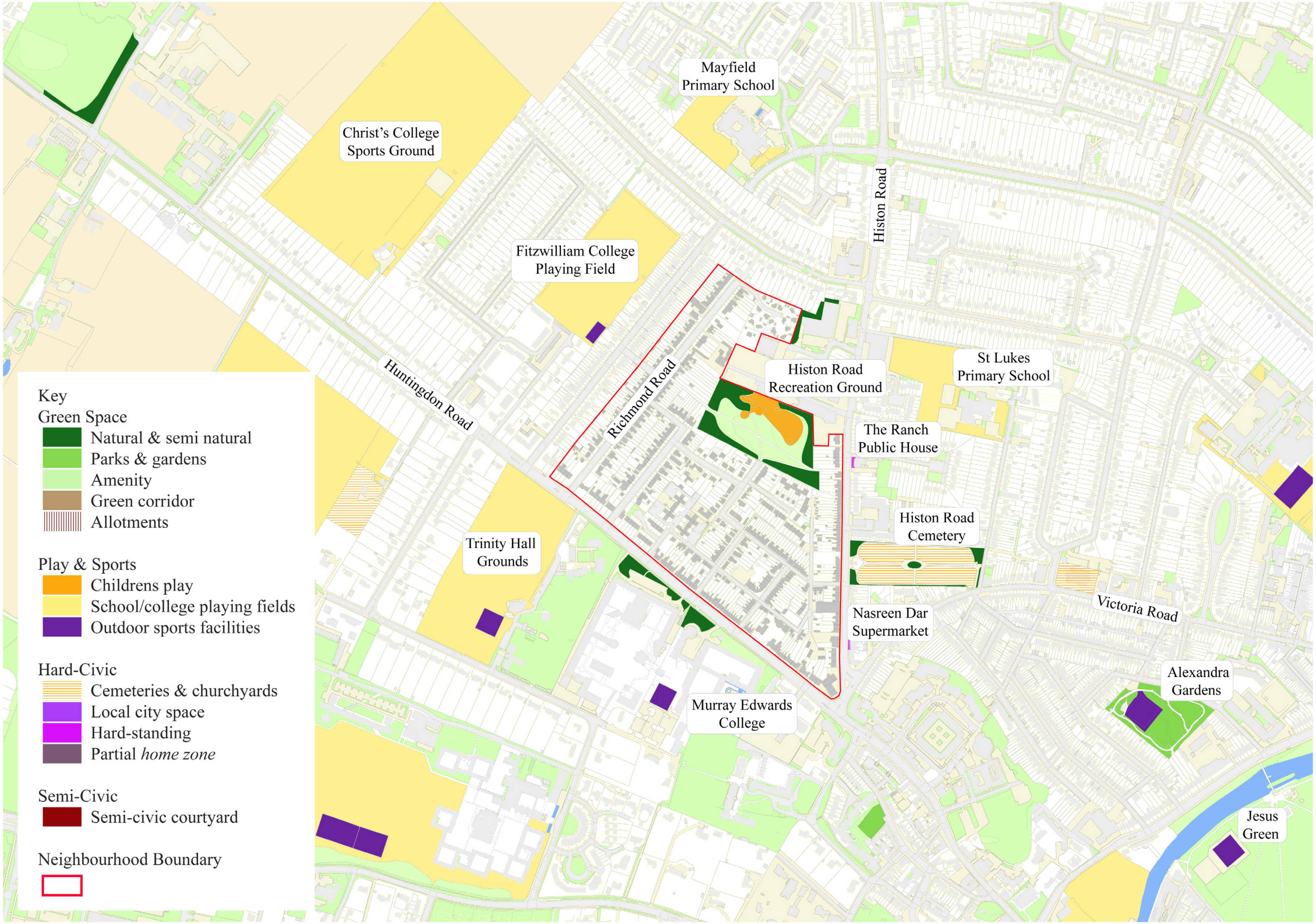

Supplement: Supplementary file 1 [file datasheet_1.pdf]
